# Insights on small molecule binding to the Hv1 proton channel from free energy calculations with molecular dynamics simulations

**DOI:** 10.1038/s41598-020-70369-4

**Published:** 2020-08-12

**Authors:** Victoria T. Lim, Andrew D. Geragotelis, Nathan M. Lim, J. Alfredo Freites, Francesco Tombola, David L. Mobley, Douglas J. Tobias

**Affiliations:** 1 Department of Chemistry, University of California, Irvine, CA 92697 USA; 2 Department of Pharmaceutical Sciences, University of California, Irvine, CA 92697 USA; 3 Department of Physiology and Biophysics, University of California, Irvine, CA 92697 USA; 4 Chao Family Comprehensive Cancer Center, University of California, Irvine, CA 92697 USA

**Keywords:** Cancer, Ion channels, Ion channels, Biophysical chemistry, Thermodynamics, Computational chemistry, Molecular dynamics, Statistical mechanics

## Abstract

Hv1 is a voltage-gated proton channel whose main function is to facilitate extrusion of protons from the cell. The development of effective channel blockers for Hv1 can lead to new therapeutics for the treatment of maladies related to Hv1 dysfunction. Although the mechanism of proton permeation in Hv1 remains to be elucidated, a series of small molecules have been discovered to inhibit Hv1. Here, we computed relative binding free energies of a prototypical Hv1 blocker on a model of human Hv1 in an open state. We used alchemical free energy perturbation techniques based on atomistic molecular dynamics simulations. The results support our proposed open state model and shed light on the preferred tautomeric state of the channel blocker. This work lays the groundwork for future studies on adapting the blocker molecule for more effective inhibition of Hv1.

## Introduction

The flux of ions across the cell membrane is regulated by a variety of ion channels. Hv1 is a voltage-dependent ion channel whose main function is to conduct protons through the cell membrane. Like voltage-dependent metal ion channels, the activation of Hv1 is regulated by voltage-sensing domains (VSDs), which are transmembrane modular units that detect changes in the membrane potential. However, in contrast to voltage-dependent metal ion channels, Hv1 lacks a separate pore domain as the VSD is sufficient for proton permeation in addition to mediating channel gating^1–3^. The action of Hv1 contributes to physiological processes such as the production of reactive oxygen species, the bioluminescence of dinoflagellates, and the maturation of human sperm^4–6^. Hv1 expression was found to be selectively enhanced in metastatic relative to non-metastatic breast tissue and inhibition of Hv1 was shown to reduce cancer metastasis and tumor development^7–9^. More generally, the development of inhibitors to block the Hv1 channel may also lead to therapeutic benefit for other Hv1-related maladies, including allergies^10,11^ or exacerbated brain damage in ischemic stroke^12^.

The full structure of human Hv1 has not yet been experimentally determined. Human Hv1 is a homodimer, but each monomer has its own pore and can function independently^13,14^. We recently developed an atomistic model of the human Hv1 VSD open state using molecular dynamics (MD) simulations on the microsecond timescale in a hydrated lipid bilayer under an applied membrane potential^15^. As discussed in Geragotelis et al.^15^, there exist other proposed structures for the human Hv1 open state^16–20^, which differ in the way they were generated or the templates they were based upon. These other structures were either modeled from crystallographic structures of voltage-gated ion channels which do not contain a native permeation pathway through the VSD^16–18,20^, or they have alternative VSD conformations from restraints applied during MD simulations^19^. Our structure was modeled starting from the crystal structure of a chimeric construct based on mouse Hv1 in a putative closed state^21^ from which we generated closed and open state configurations exclusively of the applied membrane potential. The elicited conformational changes were consistent with the mechanism of activation proposed for metal ion channel VSDs^22–24^. Further investigation of our open state model with respect to small molecule inhibitor binding would contribute to its validation and refinement and pave the way to designing more effective channel blockers.

Effective inhibition of Hv1 can be achieved by ligands that bind the extracellular side of the channel, including Zn$^{2+}$^1,2,25^; small peptide toxins such as Hanatoxin, C6, and AGAP/W38F^26–28^; or by ligands that bind the cytoplasmic side, such as guanidine derivatives^29,30^. We focus on a representative guanidine derivative, 2-guanidinobenzimidazole (2GBI), to understand how it binds to the channel. Compounds targeting the VSD often have multiple targets^31,32^. However, the open state VSD in Hv1 is more hydrated compared to non-conducting VSDs and offers enough space for 2GBI to bind at a location that, in other VSDs, is occupied by S4 arginines^33–36^. We propose that this feature could be exploited for the development of Hv1-specific drugs. 2GBI and related compounds can produce maximal inhibition of human Hv1 of > 90% and can block Hv1 both at the plasma membrane and in intracellular compartments. The binding of 2GBI to Hv1 is described as a “foot in the door” mechanism of block, by which binding of the ligand prevents channel closure and slows down channel deactivation^23^. There is currently no known experimental structure of 2GBI in complex with Hv1, and the details of 2GBI binding are not completely known; however, mutagenesis experiments^29^ suggest several key residues that play a role, including the selectivity filter D112^37^ as well as F150 and R211 lining the central constriction region^16^.

**Figure 1:**
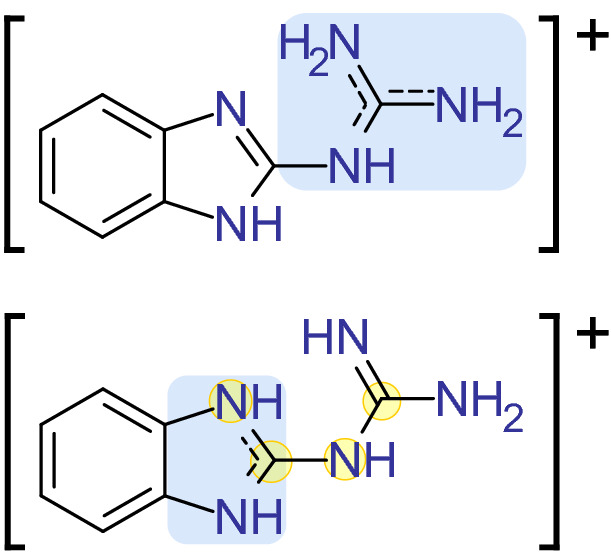
Two tautomers of 2GBI are investigated in Hv1 binding: “gbi1” (top) and “gbi2” (bottom). Blue regions have higher positive charge density. Yellow circles mark atoms of the dihedral angle selected for the dihedral scan in this work.

The structure of 2GBI (Fig. 1, top) was rationalized by the fact that Hv1 is inhibited by the guanidinium ion^13^ which is structurally similar to the voltage-sensing arginine residues in the S4 helix^38^. 2GBI is a more effective inhibitor for Hv1 than guanidinium with the addition of its benzimidazole moiety. Despite that fact, replacement of one nitrogen atom on the five-membered ring by an oxygen atom resulted in less effective inhibition than even guanidinium^23^. Details of the binding site aside, this raises the question of the structure and charge distribution of 2GBI in relation to its tautomeric forms^39^.

In this work, we studied the binding of 2GBI to our open state human Hv1 model^15^ using alchemical free energy perturbation with atomistic molecular dynamics simulations. We considered a series of six protein mutations previously shown to alter 2GBI apparent binding affinity, and we computed relative binding free energies of these mutations compared to wild type Hv1 for two tautomeric forms of the inhibitor. This work reveals structural characteristics of 2GBI binding, emphasizes the prevalence of gbi2 in binding Hv1, and may inform future efforts to optimize small molecule blockers of Hv1.

## Methods

### Ligand parameterization with CGenFF

Our study examines two tautomers of the positively charged 2GBI, depicted in Fig. 1. Both of these structures contain a guanidine-like moiety but have different centers of excess charge, located in either the guanidine region (gbi1) or in the central imidazole moiety (gbi2). The force field for each tautomer was developed using the CHARMM General Force Field (CGenFF)^15,40,41^. We generated reference structures from gas phase quantum mechanical (QM) geometry optimizations using the Psi4 software package^42^. We employed the MP2/6-31G* method^43–47^ to stay consistent with CGenFF development^41^. The generated gbi2 force field was modified to achieve better agreement regarding the geometry-optimized structures between the force field and QM results. The force field modification details and final parameters for both ligands can be found in the supplementary information (“Ligand parameterization for 2GBI”).

### Docking calculations and pose refinement with molecular dynamics simulations

We docked each tautomer of 2GBI into Hv1 with AutoDock Vina version 1.1.2^48^. Twenty protein configurations were selected from a ~ 33 μs simulation^15^, and we removed the membrane and all water molecules during docking. While the presence or absence of water molecules may influence results from docking^49,50^, specific water molecules involved in binding 2GBI are as of yet unknown. Thus, our aim in docking was just to generate an initial set of binding poses to be further refined in MD simulations, which allow waters to rearrange as they prefer. The ligand dihedral angle, defined by the four atoms marked in Fig. 1 (bottom), was held fixed in a planar conformation to prevent high-energy initial structures resulting from strained non-planar conformations (see Supplementary Information Figs. S1, S4).

The resulting poses were filtered by a “reverse clustering” technique. We used the Clustering plugin^51^ in VMD^52^ to cluster all poses by root mean square deviation (RMSD) with a 3.0 Å cutoff. We retained poses that were distinct from each other by more than this threshold. This process yielded a diverse set of starting poses ensuring good coverage of the multiple possible binding modes in the binding site, allowing minor differences between similar poses to be explored by our subsequent MD simulations. Each binding pose was placed back into its hydrated protein-membrane configuration, and we removed water molecules overlapping within 2 Å of the ligand, otherwise retaining original hydration of the pore.

We then ran short (5 ns) MD simulations for all distinct poses of each 2GBI tautomer using the NAMD software package, version 2.11^53^. Prior to dynamics, 5000 minimization steps were performed using the conjugate gradient algorithm. Backbone alpha carbon atoms were fixed during minimization using harmonic position restraints with a force constant of 1 kcal/mol/Å$^2$. The restraints were gradually turned off over 400 ps. Dynamics were then run with a 2 fs time step in the isothermal-isobaric ensemble at a temperature of 300 K and a pressure of 1 bar. The Langevin thermostat was applied with a damping constant of 5 ps$^{-1}$, and the Langevin piston method was applied with an oscillation period of 200 fs and a damping time of 100 fs. Periodic boundary conditions were applied in all dimensions, with the *z*-axis normal to the membrane bilayer. The SHAKE algorithm was used to restrain all bonds involving hydrogen atoms to their equilibrium values. Long-range electrostatic interactions were applied with the Particle Mesh Ewald (PME) algorithm; short-range Lennard-Jones and Coulombic interactions were calculated using a cutoff of 12 Å and a switching function applied beyond 10 Å. Bonded interactions and short-range forces were calculated every 2 fs, and long-range forces were calculated every 4 fs. An electric field was applied using a field vector of 0.14 kcal/(mol Å e) in the *z* direction, corresponding to a depolarizing membrane potential of +150 mV.

The final poses selected for free energy calculations were chosen based on the stability of the ligand in the binding position as well as evaluation of the hypothesized placement of 2GBI from experimental double-mutant cycle analyses^29^. Our evaluation criteria for pose analysis is outlined in the supplementary information (“Pose selection and refinement with MD”). The poses were equilibrated for at least 20 ns before initiating free energy calculations. We verified that the pore retained hydration in each of the docked configurations during the equilibration simulations (see Supplementary Information Fig. S6).

Configurations from our MD simulations for pose refinement and subsequent free energy calculations were all rendered using VMD version 1.9.3^52^.

### Alchemical free energy calculations

**Figure 2:**
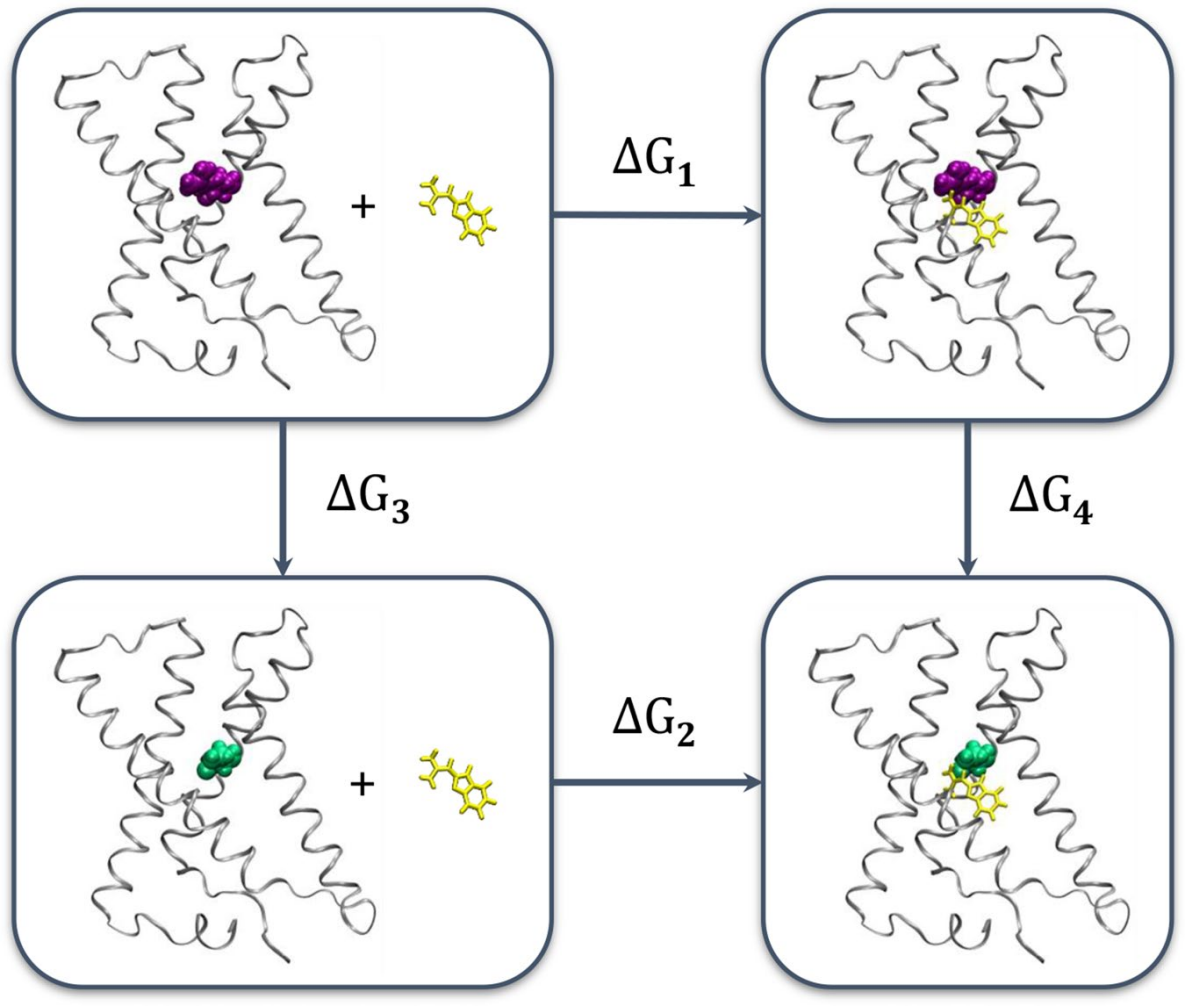
Thermodynamic cycle used to compute relative binding free energies. The initial side chain from wild type Hv1 is shown in purple vdW spheres, and the final side chain after alchemical mutation of Hv1 is shown in green vdW spheres. The 2GBI ligand is drawn in yellow licorice. The membrane and water molecules are not shown for clarity. The relative binding free energy from experiment is calculated from $\Delta \Delta G = (\Delta G_2 - \Delta G_1)$, which is thermodynamically equivalent to the *alchemically* computed relative binding free energy of $\Delta \Delta G = (\Delta G_4 - \Delta G_3)$.

We applied the thermodynamic cycle approach as depicted in Fig. 2 to calculate the change in the binding free energy of 2GBI from wild type Hv1 to the following protein mutations: D112E, V178A, S181A, V109A, R208K, and R211S. The first and last single letter codes denote the starting and final amino acids, respectively. The relative binding free energy in this cycle is calculated as the difference in the free energies of mutation from the holo state to the apo state, i.e., $\Delta \Delta G = (\Delta G_4 - \Delta G_3)$. The computed values were evaluated alongside the relative binding energies from experimental mutagenesis studies^29^, which correspond to processes $\Delta G_1$ and $\Delta G_2$ of the thermodynamic cycle; i.e., $\Delta \Delta G = (\Delta G_2 - \Delta G_1)$.

Processes $\Delta G_3$ and $\Delta G_4$ of the thermodynamic cycle represent the alchemical transformations conducted computationally. The initial and final states for each of $\Delta G_3$ and $\Delta G_4$ are connected through a series of non-physical intermediate states (“$\lambda$ windows”) that comprise the alchemical transformation. This transformation is controlled by a parameter $\lambda$, starting from a value of zero at the initial state (i.e., wild-type protein residue) and reaching a value of one at the final state (i.e., mutant protein residue).

The alchemical transformation is subdivided into 40 equivalently spaced $\lambda$ windows, such that the first window ranges from $\lambda =0$ to $\lambda =0.025$ and the final window ranges from $\lambda =0.975$ to $\lambda =1$. With simple linear modification of interactions, “end point catastrophes” may result when ($\lambda \rightarrow 0$) or ($\lambda \rightarrow 1$), during which incoming atoms may appear where other particles already exist^54^. We avoid this by applying a soft-core potential on perturbed atoms to gradually scale their short-range nonbonded interactions with the rest of the system^55^.

Each alchemical transformation was conducted in both the forward and reverse directions (e.g., aspartate mutated to glutamate as well as glutamate mutated to aspartate) to facilitate convergence and sampling of overlapping phase space of the end states. While the free energy difference between two states can be determined by the Zwanzig relationship^56^, this method is often slow to converge and results in poor phase space overlap^57,58^. The Bennett acceptance ratio (BAR)^59^ minimizes the statistical variance between two ensembles with overlapping configurational space and yields the optimal averaging of the forward and reverse simulations. We used BAR to combine the data from both directions to estimate the final free energies for each of $\Delta G_3$ and $\Delta G_4$. The reported uncertainties of the relative binding free energies are the root sum square values of the $\Delta G_3$ and $\Delta G_4$ standard errors from BAR.

A dual-topology paradigm was applied throughout the alchemical transformation in which both the initial and final states were simultaneously present but non-interacting. Only nonbonded interactions for perturbed (incoming or outgoing) atoms contributed to the cumulative free energy. Van der Waals interactions were scaled as a function of $\lambda$ across the full range from zero to one. Electrostatic interactions of the annihilating atoms were linearly decoupled over the first half of the transformation ($\lambda =0$ to $\lambda =0.5$), after which electrostatic interactions were linearly coupled for the incoming atoms.

We computed the relative binding free energies using alchemical free energy perturbation with atomistic molecular dynamics simulations in NAMD^53^. Each window started from the same initial hybrid structure and comprised 1,000 minimization steps, 1 ns of equilibration, and 4 ns of production. The MD simulation settings were maintained as described earlier. For each protein mutation, we simulated a total of 400 ns, consisting of 5 ns in each of the 40 $\lambda$ windows for the forward and reverse directions. The data from each $\lambda$ window was subsampled to extract uncorrelated, effectively independent samples using the pyMBAR Python package^60^. For all apo/holo states, ligand tautomers, and protein mutations represented in this study, including mutation S211R (see “Results and discussion” section), the total simulation time is 8.4 μs.

We modified our standard setup for free energy calculations for mutations R208K and R211S. In these two cases, we additionally applied flat-bottom distance restraints to prevent hypermobility of the arginine and lysine residues. During normal MD simulations, these positively charged side chains are involved in salt bridge interactions with nearby acidic residues. Residue 208 is in proximity to D119, D123, and E192, and residue 211 is in proximity to D112 and D185. However, these salt bridges are not able to be maintained during the alchemical transformations because of the decoupled nonbonded interactions in the dual-topology hybrid molecule. In other words, considering the case of R208K when arginine is mutated to lysine, arginine is fully interacting and present at the start, when $\lambda =0$. In the final $\lambda$ stage when $\lambda =1$, arginine is now non-interacting with the system and therefore does not form its usual contacts. The non-interacting, flexible side chains end up sampling an artificially broadened area of phase space. This improperly skews the phase space overlap between the forward and reverse calculations and adversely affects the free energy estimate (see “Results and discussion” section). To avoid this issue and improve convergence of the calculations, we added additional distance restraints on the flexible mutating residues for R208K and R211S. The flat-bottom restraints were applied from the terminal nitrogen atom in arginine or the terminal carbon of lysine to surrounding protein residues that were not involved in the alchemical transformation. The reference distances were chosen to be large enough such that the restraint energies were zero when applied to the initial or final structure yet restrictive enough to prevent excessive side chain mobility during the free energy calculations (see supplementary information: “Flat-bottom restraints for mutations involving Arg or Lys residues”). The force constants on the restraint walls were set to 10 kcal/mol/Å.

Because some of our free energy calculations involved changes in formal charge, it was necessary to take particular care in these cases. Specifically, following the free energy simulations, we accounted for the energetic contribution arising from the change in net charge in the R211S mutation. To do this, we applied analytical corrections for the electrostatic finite-size effects as described by Rocklin et al.^61^. That being said, these corrections were found to have almost no impact on the final relative binding free energies as we show in the "Results and discussion" section.

## Results and discussion

**Figure 3:**
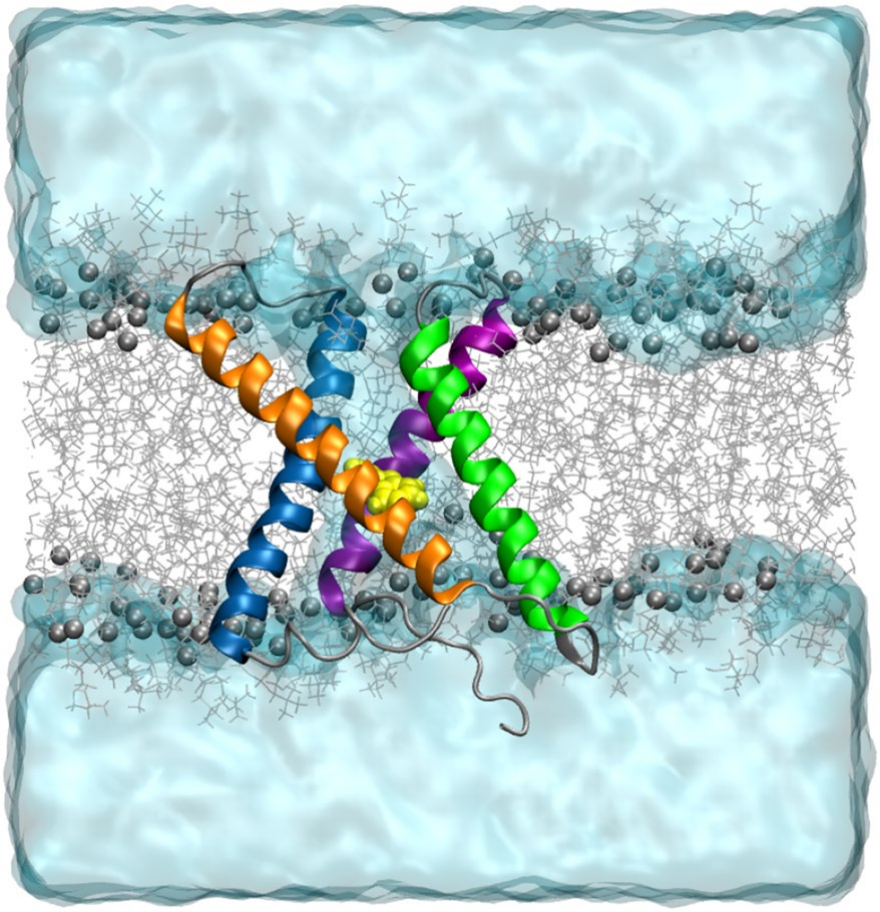
Configuration snapshot of 2GBI (gbi1) bound to Hv1, rendered in VMD^52^. The four alpha helical segments of Hv1 are colored as follows: S1 in blue, S2 in orange, S3 in green, and S4 in purple. 2GBI is shown in yellow with the VDW representation. Waters are drawn in slabs of light blue, and lipids are depicted as gray sticks with their carbonyl carbons shown as vdW spheres. The extracellular region is on top.

We employed docking calculations, atomistic molecular dynamics simulations, and alchemical free energy calculations to examine the binding of 2GBI to human Hv1 (Fig. 3). We computed relative binding free energies for a series of six protein mutations compared to wild type Hv1. Each set of binding free energy calculations was conducted for two tautomers of 2GBI, gbi1 and gbi2, which vary in their centers of excess charge (Fig. 1). Our results show general qualitative agreement with experimental mutagenesis data and suggests that one tautomer may be more relevant than the other in the protein-bound configuration. We also discuss some limitations when carrying out alchemical transformations of flexible protein side chains.

### Positioning of 2GBI tautomers within the Hv1 open state

From the docking calculations, we employed a “reverse clustering” approach to identify 2GBI binding poses (see Methods). That is to say, because the binding mode is unknown and given that docking does not predict definitive binding modes, we aimed to sample a variety of potential poses of 2GBI within the expected binding region in order to find the most reasonable binding mode. The resulting poses from docking calculations were assessed using the predicted interactions between 2GBI and Hv1 from experimental mutagenesis data^29^. Hong et al. proposed a model of binding involving residues D112, F150, S181, and R211. For both gbi1 and gbi2, we selected binding poses where 2GBI was in close enough proximity to interact with these residues. Based on these poses, we note likely contacts with two additional residues, D185 and F182.

**Figure 4:**
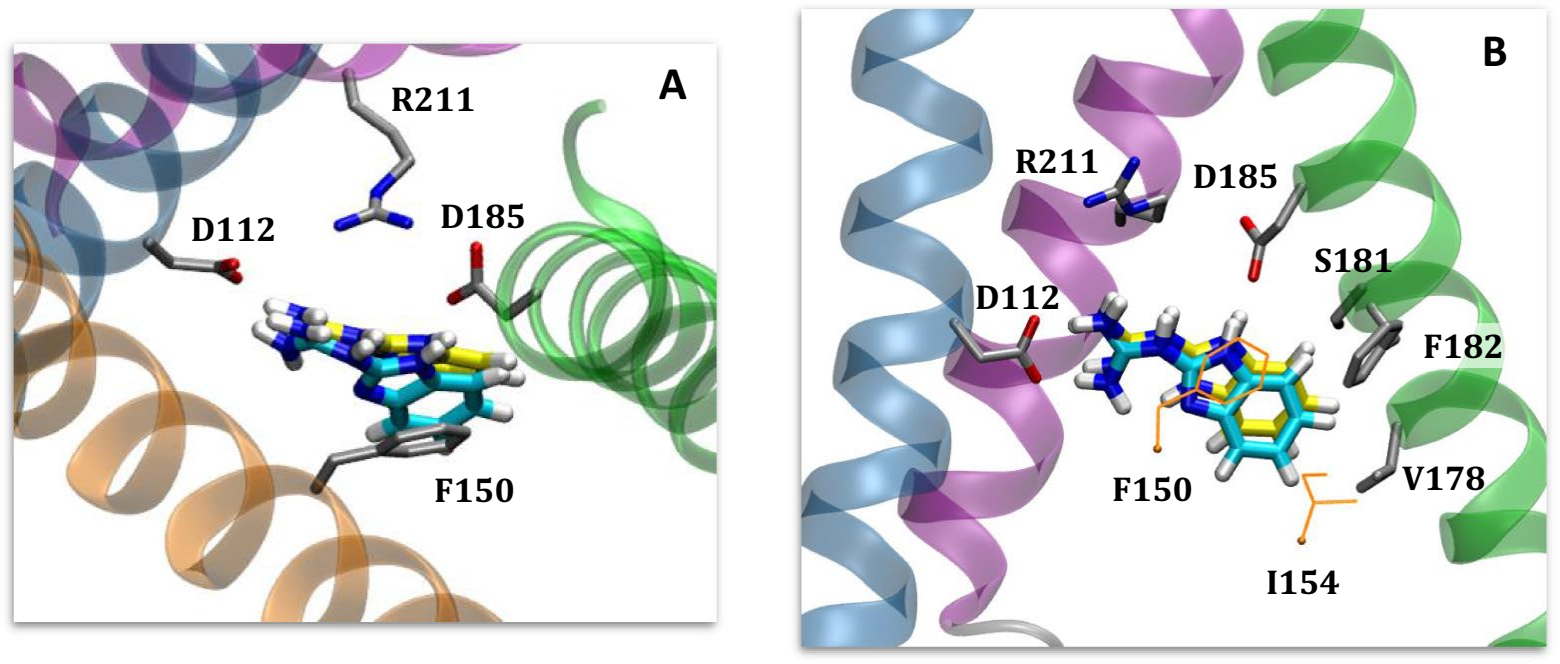
2GBI docked into the open state model of human Hv1, rendered in VMD^52^. The binding pose is consistent with experimental mutagenesis data. Segments are colored in the same manner as in Fig. 3. The ligand is colored cyan for gbi1 and yellow for gbi2. (**A**) Top-down view from the extracellular end of the channel of bound 2GBI. Loops are hidden for viewing clarity. (**B**) Lateral view of Hv1 (extracellular side on top) with S2 hidden for clarity. The two residues F150 and I154 are part of S2.

Following refinement in MD simulations, we selected a binding pose (Fig. 4) with the following features: interactions of the guanidine region with the charged residues D112 and R211; hydrogen bond donation from one of the imidazole NH groups to acidic residue D185; and proximity of the hydrophobic residues F150, I154, and F182 to the benzimidazole moiety. While D112 and R211 are believed to interact in the open state conformation of Hv1^62^, the presence of 2GBI as a channel blocker in the proposed binding site necessarily interrupts this salt bridge formation. We chose the same binding pose for gbi1 and gbi2.

Our pose is distinct from that proposed in recent work by Gianti et al.^19^ who performed induced fit docking of 2GBI (our gbi1 structure). They obtained a binding pose in which 2GBI is roughly in the same region of Hv1 but angled differently such that the benzo moiety of 2GBI is closer to S1 than to S3. The location of the guanidine group in our pose pointing to R211 is more consistent with experimental work^29^. Differences in binding configuration may be in part due to differences in our open state models^15^.

Chamberlin et al. also described a binding site for 2GBI, using the ligand model we refer to as gbi1, docked into a model of *Ciona intestinalis* Hv1, which itself was based on the crystal structure for the open state chimeric channel Kv1.2-2.1^18,63^. Their ligand position is similar to our results in that the 2GBI benzo moiety is adjacent to F150; however, their binding pose has the guanidine moiety of 2GBI pointing towards the intracellular end of the channel in contact with E153, D174, and E171. While the salt bridge patterns are similar between both protein models, the Chamberlin et al. protein structure has S2 in a relatively higher position (towards extracellular side) than the other segments. Protein models notwithstanding, this intracellular-pointing ligand orientation would preclude any interactions with S181 or R211 as proposed by Hong et al.^29^ (see Supplementary Information Fig. S7). Neither of Gianti’s or Chamberlin’s studies considered alternate tautomers or binding free energy calculations.

### Calculation of relative binding free energies

**Figure 5:**
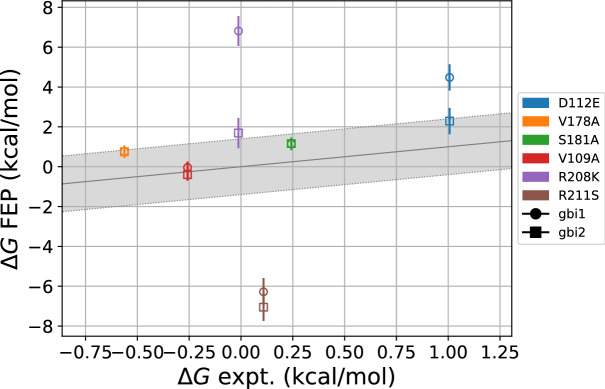
Relative binding free energies for the two 2GBI tautomers and six protein mutations considered. The error bars represent the root sum square values of standard errors from the Bennett acceptance ratio for the apo and holo states. The shaded region represents ±1.4 kcal/mol from the experimental value, representing a 10-fold change in the equilibrium constant. Alternate tautomers use different symbols. The markers for V178A (orange) and S181A (green) overlap for gbi1 and gbi2. Computed relative binding free energies are in qualitative agreement with experimental data, especially for gbi2.

We calculated the relative binding free energies for two tautomers of 2GBI binding to wild type and mutant Hv1 using our open state model. The relative binding free energies were computed using molecular dynamics simulations with alchemical free energy perturbation. We considered six Hv1 mutations, previously reported by Hong et al. using electrophysiological and site-directed mutagenesis experiments^29^. Compared to 2GBI binding to wild type Hv1, these mutations comprise the effects of unfavorable binding (D112E, S181A), favorable binding (V178A, V109A), and relatively no change in the binding free energy (within ~0.1 kcal/mol: R208K, R211S). These values are plotted in Fig. 5 and listed in Table 1. Overall, with the exception of R211S, the calculated values are in good qualitative agreement with the experimental mutagenesis data which contributes to the validity of our open state Hv1 model. Between the two 2GBI tautomers, the results for gbi2 are more consistent with experiment. The mean absolute error (MAE) for gbi2 is 2.1 kcal/mol, compared to gbi1 with an MAE of 3.2 kcal/mol, which suggests that gbi2 may be the more prevalent species. Without consideration of R211S (discussed later), the MAE improves to 1.1 kcal/mol for gbi2 and 2.5 kcal/mol for gbi1.

**Table 1:** Binding free energies of 2GBI (gbi1 and gbi2) to Hv1, for mutant Hv1 compared to wild type Hv1. The uncertainties represent the root sum square values of standard errors from the Bennett acceptance ratio for the apo and holo states. The data presented here is also plotted in Fig. 5.

mutation	expt	gbi1	gbi2
D112E	$1.01 \pm 0.05$	$4.9 \pm 0.7$	$2.3 \pm 0.7$
V178A	$- 0.56 \pm 0.05$	$0.8 \pm 0.3$	$0.8 \pm 0.3$
S181A	$0.24 \pm 0.05$	$1.1 \pm 0.3$	$1.1 \pm 0.3$
V109A	$- 0.26 \pm 0.06$	$0.0 \pm 0.3$	$- 0.4 \pm 0.3$
R208K	$- 0.01 \pm 0.05$	$6.8 \pm 0.8$	$1.7 \pm 0.8$
R211S	$0.11 \pm 0.05$	$- 6.3 \pm 0.7$	$- 7.1 \pm 0.7$

Protein mutations which involve charged side chains (D112E, R208K, and R211S) are more challenging for which to compute binding free energies compared to mutations having neutral residue end states. D112E involves charge movement by the addition of a methylene group, R208K changes the charge density of the cationic region^64^, and R211S changes the charge of the system from neutral to −1 *e*. There are greater disparities in the relative binding free energies of gbi1 and gbi2 for these three mutations, with gbi2 values generally closer to experiment.

**Figure 6:**
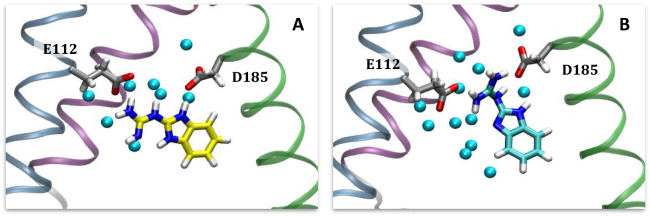
2GBI bound to the human Hv1 D112E mutant in the (**A**) gbi2 and (**B**) gbi1 systems, rendered in VMD^52^. The ligand and surrounding residues are displayed in licorice representation, and the oxygen atoms of waters within 3 Å of the ligand are shown as light blue spheres. Concerning hydrogen bonding interactions between 2GBI and the neighboring acidic residues E112 and D185, the tautomer gbi1 does not contain a protonated imidazole region as in gbi2 (see Fig. 1). It seems for this reason that gbi2 maintains its original binding pose after mutation, while gbi1 reorients in order to maximize the interactions of its more positively charged guanidine moiety with both E112 and D185.

Compared to WT, the reported experimental value for the D112E mutation leads to a change in binding free energy of +$1.01 \pm 0.05$ kcal/mol. In our simulations, this mutation induced a shift in the gbi1 ligand orientation after ~5 ns as gbi1 rotates to prevent crowding of the glutamate residue and to maximize interactions of its guanidine moiety (Fig. 6). Lengthening of the side chain of residue 112 appears to encroach on the binding site and affect the native hydrogen bonding interactions between the positively-charged 2GBI and the nearby acidic side chains of residues 112 and 185 (see Supplementary Information Fig. S8). This crowding may explain the unfavorable binding represented by the relative binding free energy.

Of the mutations considered here, the V178A mutation is most favorable for binding 2GBI, with an experimental relative binding free energy of $- 0.56 \pm 0.05$ kcal/mol compared to wild type. In contrast, our predicted value was between 0.7 and 0.8 kcal/mol for both tautomers, indicating binding becomes less favorable. We wondered if the experimental value reflected additional protein rearrangements not captured in the timescale of our MD simulations. To explore this further, we focused on a nearby aromatic residue, F182, in proximity to both V178 and the benzo moiety of 2GBI. This residue has been found to play a role in 2GBI binding via $\pi$-stacking interactions^65^. We hypothesized that F182 may reorient itself, occupying some of the space from the mutation of valine to alanine, in order to better interact with the bound ligand. We evaluated the energetic contribution of this putative conformational change by computing the potential of mean force for rotating the F182 $\chi _1$ dihedral angle. Our results did not support this theory but rather suggested an energetic cost of around 8 kcal/mol for the F182 aromatic ring being in a parallel configuration with the 2GBI aromatic moiety (see Supplementary Information “Potential of mean force calculation for F182 rotation after V178A mutation”).

### Hysteresis in free energy calculations involving “floppy” residues

**Figure 7:**
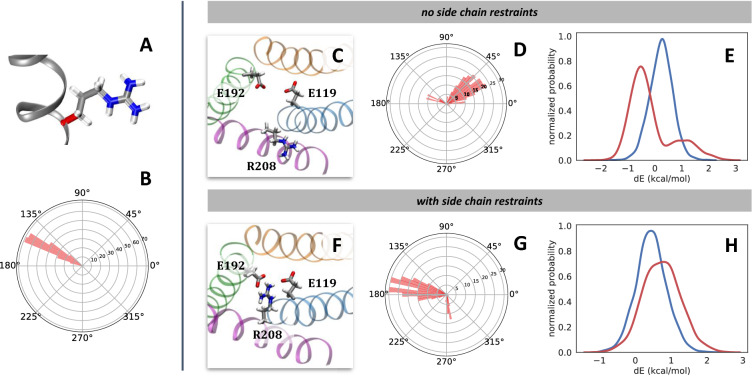
Correlation of the mutating arginine $\chi _1$ dihedral angle to the work overlap in the R208K transformation. Without side chain restraints, $\chi _1$ takes unexpected values, causing the side chain to point outside the Hv1 channel and leading to poor overlap of forward and reverse work values. (**A**) The central bond of the arginine $\chi _1$ dihedral angle is drawn in red. (**B**) Histogram in polar coordinates of dihedral angle values belonging to R208 $\chi _1$ during a normal, fully-interacting MD simulation. (**C**) View of the channel from the extracellular side. The incoming K208 residue is not shown for clarity. (**D**) Polar histogram of dihedral angle values belonging to R208 $\chi _1$ during alchemical free energy calculation of R208K. The angle values are plotted from the $\lambda =[0.400, 0.425]$ window. (**E**) Overlap of forward (blue) and reverse (red) work values for the $\lambda =[0.400, 0.425]$ window in the R208K mutation of the apo protein. Both R208 and K208 partially interact with the system as per the alchemical potential. (**F**–**H**) Analogous to (**C**–**E**), respectively, but with the addition of flat-bottom distance restraints.

One way that we evaluate convergence in our free energy calculations is by analyzing the histogram overlap of work values from the forward and reverse processes^66^. We observe that mutations involving flexible and charged side chains, such as arginine and lysine, are more prone to have poor overlap, signifying inadequate sampling of overlapping regions of phase space during the alchemical transformations. This presumably arises from the scaling of nonbonded interactions. Towards the start of the transformation ($\lambda$ close to 0), the incoming atoms are mostly decoupled from, or not interacting with, the rest of the system. Similarly, at states where $\lambda$ is close to 1 near the end of the alchemical transformation, the outgoing atoms are decoupled from the rest of the system. The decoupled nonbonded interactions may “free” the flexible, charged side chains to adopt various nonphysical conformations.

We illustrate this hysteresis in context of the R208K Hv1 mutation, focusing on the arginine residue. In wild type Hv1, the measure of the R208 $\chi _1$ dihedral angle (Fig. 7A) averaged around 154$^{\circ }$ in equilibrium simulations (histogrammed in Fig. 7B), and R208 is in close proximity to the acidic side chains E119 and E192. However, during free energy calculations with the scaled nonbonded interactions, the side chain pivots such that R208 is artifactually outside the channel (Fig. 7C), and the $\chi _1$ dihedral angle values are inconsistent with the range expected from wild type Hv1 simulations (Fig. 7D). The lack of fully coupled van der Waals and electrostatic interactions of the floppy, charged residue with the rest of the system interrupts native ionic interactions and leads to unnatural wandering of the side chain in simulations of these artificial intermediate states. The sampled unphysical configurations were directly correlated with poor overlap between the forward and reverse transformations, evidenced here by bimodal histograms of the work values from the forward and reverse transformations (Fig. 7E). Similar observations were made for the lysine $\chi _1$ dihedral angle.

We reduced this hysteresis by imposing a series of flat-bottom distance restraints with nearby interacting acidic residues (see Methods and supplementary information: “Flat-bottom restraints for mutations involving Arg or Lys residues”). This yielded fewer abnormal side chain configurations (Fig. 7F–G) and better overlap of the forward and reverse work (Fig. 7H).

### Further exploration on R211S

**Figure 8:**
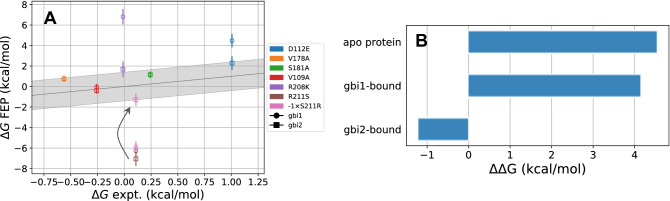
Calculated relative binding free energies for the mutation of residue 211 between the arginine and serine end states reveal discrepancies based on the starting structure. (**A**) Computed relative binding free energies, analogous to Fig. 5, with the addition of the S211R mutation. The pink markers show the negative of the S211R values to compare on the same scale as R211S. The R211S relative binding free energies ideally should be the same as those for $- 1 \times$ S211R. The error bars represent the root sum square values of standard errors from the Bennett acceptance ratio for the apo and holo states. (**B**) Bar plot showing the change in the computed $\Delta G_3$ (apo protein) or $\Delta G_4$ (holo protein) of individual processes of the thermodynamic cycle (Fig. 2) for the R211S/S211R mutation. The apo protein value represents $\Delta G_{3,\> R211S} - (- 1 \times \Delta G_{3,\>S211R})$, and the analogous expression is applied to the $\Delta G_4$ values of gbi1 and gbi2. These values should be zero since free energy is a state function which is independent of the path taken.

The computed binding free energies of R211S are least consistent with experimental results. Our results were predicted to be overly favorable by −6.3 kcal/mol for gbi1 and −7.1 kcal/mol for gbi2 (the experimental reference value is 0.1 kcal/mol). We considered whether a potential contribution may have been the change in total charge of the system. The mutation from a positively charged arginine to a neutral serine changes the system’s net charge from neutral to −1 *e*. Our simulations employed the particle-mesh Ewald (PME) method for treatment of long-range electrostatics in the periodic system. However, PME enforces neutrality by introducing a uniform neutralizing background charge. Therefore, with the non-neutral end state, the calculated free energy not only has the expected contribution of turning off the arginine charge but also undesired contribution for turning on the neutralizing background charge. We corrected for the electrostatic finite-size effects by applying an analytical correction based on the Poisson–Boltzmann continuum electrostatics model in CHARMM (version c40b1)^61,67^. The corrections to the binding free energy were not trivial when considered individually (apo = −3.1042 kcal/mol, gbi1 = −3.1030 kcal/mol, gbi2 = −3.1019 kcal/mol). However, they become negligible when considering relative binding free energies. As a result, we believe that the change in net charge is not the primary reason for poor agreement of the experimental and computed R211S relative binding free energies.

We investigated the possibility of unconverged conformational sampling as a cause for the R211S outlying behavior. We performed the same transformation in the opposite direction, i.e., preparing the system with S211 and perturbing to R211. The S211 structures for the apo, gbi1-bound, and gbi2-bound states came from the end of the respective R211S transformations. Each configuration was equilibrated for 5 ns before the S211R free energy calculations. We maintained the same $\lambda$ schedule and simulation parameters as described earlier.

We consider the negative value of S211R in order to adequately compare the free energy change in the same direction, and we subsequently refer to this mutation as $- 1 \times \mathrm {S211R}$. The free energies for R211S and $-1 \times \mathrm {S211R}$ should be the same ideally, as free energy is a state function which is independent of the path or direction taken between two states. The computed $- 1 \times \mathrm {S211R}$ binding free energies for gbi1 and gbi2 are −5.9 kcal/mol and −1.3 kcal/mol respectively (Fig. 8A, brown vs. pink). In this plot, gbi1 seems to be more consistent between R211S and $- 1 \times \mathrm {S211R}$. However, the plotted values are relative binding free energies where the apo value is subtracted from each of the gbi1 and gbi2 values (see the thermodynamic cycle in Fig. 2). Here, the $\Delta G_3$ of apo and the $\Delta G_4$ of gbi1 increase by about 4 kcal/mol between R211S and $-1 \times \mathrm {S211R}$, but the $\Delta G_4$ for gbi2 only changes by about 1 kcal/mol between R211S and $-1 \times \mathrm {S211R}$ (Fig. 8B).

**Figure 9:**
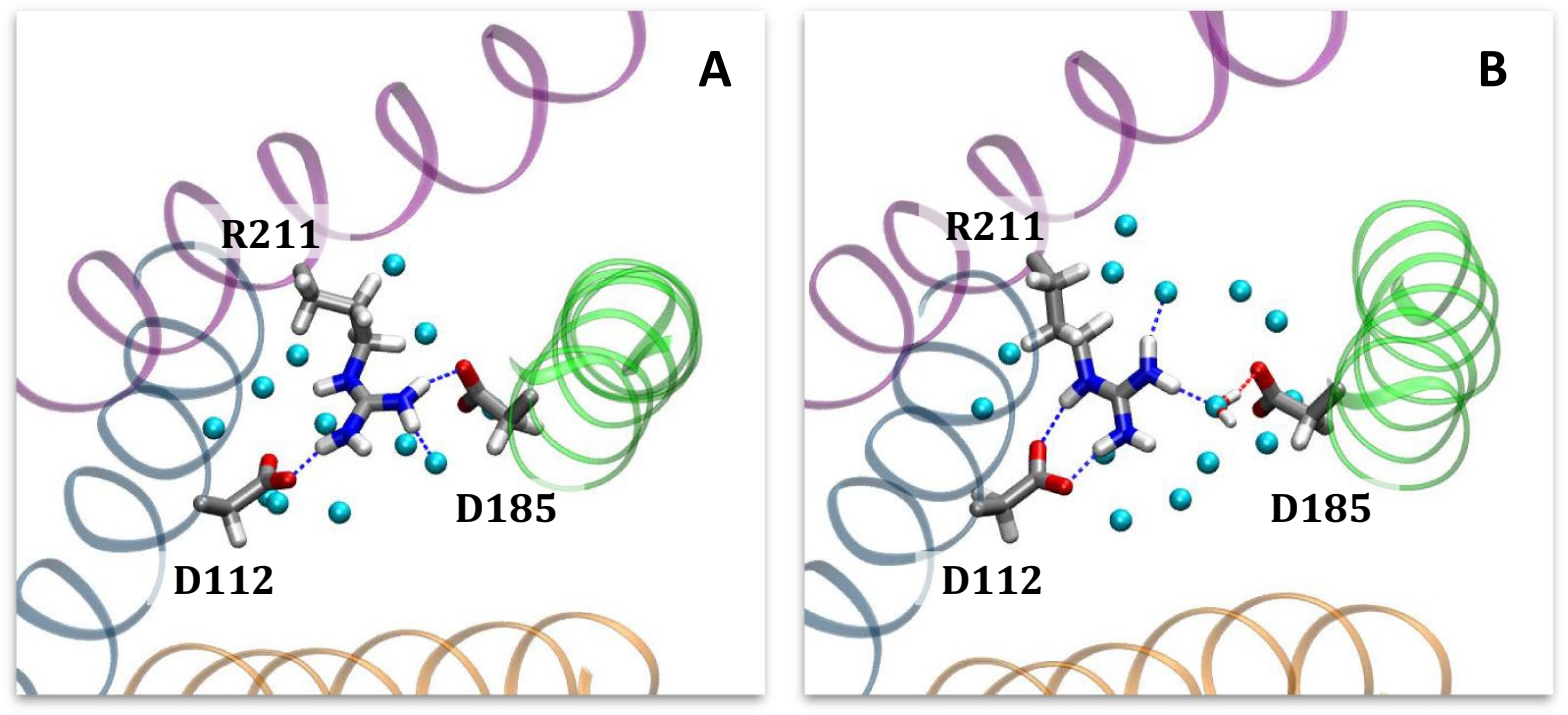
View from the extracellular side of the apo channel showing representative interaction networks around R211 (**A**) before and (**B**) after simulating a null transformation of R211 to Ser and back to Arg. Oxygen atoms of waters within 2 Å of the R211, D112, and D185 are colored as light blue spheres. The interaction differences between (**A**) and (**B**) reveal potential sampling barriers in what ought to be equivalent systems. Figures rendered using VMD^52^.

**Figure 10:**
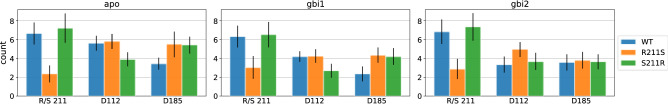
Average number of water molecules within 3 Å of the mutating residue (Arg or Ser 211), D112, and D185. The error bars represent standard deviations. Hydration patterns are not always consistent in the vicinity of R211 during the R211S and S211R mutations. For example, in the apo state, the number of water molecules near D185 increases (left subplot, right group, blue $\rightarrow$ orange bars). When mutating the system with S211 back to R211, the number of water molecules decrease near D112 instead of near D185 (left subplot, middle group, orange $\rightarrow$ green bars). It might be expected that the blue bars (wild type protein with R211) and the green bars (twice mutated protein also with R211) should be the same in all three systems. Between the two R211 states of before and after mutation, both the apo and gbi1 systems differ by 1–2 water molecules in the specified vicinity of D112 and D185, whereas gbi2 is more consistent in hydration around the two acidic residues.

We posit that this discrepancy may arise from differences in R211 interactions with other protein residues as well as differences in hydration patterns in the local region. In simulations of wild type Hv1, R211 is a hydrogen bond donor to two aspartate residues, D112 and D185 (Fig. 9A). These interactions are disrupted when the arginine is mutated to serine, and there is a slight influx of waters to the mutation site to stabilize D185. The S211R mutation reintroduces arginine, but R211 does not reform native contacts with D185, now surrounded by the water molecules (Fig. 9B). This may occur if the system is slower to dispel waters from the pore than it is to allow waters inside, especially near the buried region of the mutated residue (Fig. 10). Hence the underlying end states of R211 are not the same for the R211S and S211R mutations, potentially due to insufficient rearrangement of the surrounding solvent network. In other words, non-overlapping ensembles are being sampled in the R211S and S211R transformations, precluding reasonable comparison of these calculations with experimental mutagenesis results. Thus, our results for this transformation are reported here, but the apparent agreement between $\Delta \Delta G_{calc}$ and $\Delta \Delta G_{expt}$ for gbi2 and the $- 1 \times \mathrm {S211R}$ mutation (Fig. 8A) cannot be used meaningfully to assess the validity of our structural model due to the difficulties with this particular calculation.

## Conclusions

In this study, we employed free energy calculations with atomistic molecular dynamics simulations to characterize the binding of 2GBI to Hv1 as a foundational step to optimize small molecule blockers of Hv1. Our molecular insights of the binding mechanism of 2GBI to Hv1 agree with double-mutant cycle experiments, helping to validate our structural model for the channel and the bound state.

The mutations that were most computationally challenging were those involving charged side chains: D112E, R208K, and R211S. The R211S calculation might be further examined using enhanced sampling methods such as REST2^68–70^, with the inclusion of protein side chains and water molecules in the vicinity of R211.

Results from alchemical free energy calculations show that both the gbi1 and gbi2 tautomers are comparable in most relative binding free energies calculated. The gbi1 structure is limited in its extent of forming protein-ligand contacts without the protonated imidazole region. We reason that gbi2, the tautomer with excess charge in the imidazole region, is likely the primary state to bind Hv1. We hope to investigate the extent to which free energy calculations may be used to design new compounds based on the 2GBI scaffold, particularly with an imidazole-based pharmacophore with substitutions at the *2*-position that stabilize ring protonation, for more effective binding to Hv1 for potential therapeutic benefit.

## Supplementary Information

Supplementary Information
