# Supplementary material for: Insights on small molecule binding to the Hv1 proton channel from free energy calculations with molecular dynamics simulations
Source: Sci Rep. 2020 Aug 12;10:13587. doi: 10.1038/s41598-020-70369-4 (PMC7423955; doi:10.1038/s41598-020-70369-4)
Supplement: Supplementary file 1 — Supplementary Information [file 41598_2020_70369_MOESM1_ESM.pdf]

Supplementary Information

Insights on small molecule binding to the Hv1 proton channel from free energy calculations with molecular dynamics simulations

Victoria T. Lim[1], Andrew D. Geragotelis[1], Nathan M. Lim[2], J. Alfredo Freites[1], Francesco Tombola[3,4], David L. Mobley[1,2,*], and Douglas J. Tobias[1,*]

[1]Department of Chemistry, University of California, Irvine, California 92697, United States
[2]Department of Pharmaceutical Sciences, University of California, Irvine, California 92697, United States
[3]Department of Physiology and Biophysics, University of California, Irvine, California 92697, United States
[4]Chao Family Comprehensive Cancer Center, University of California, Irvine, California 92697, United States
[*]dmobley@mobleylab.org, dtobias@uci.edu

Ligand parameterization for 2GBI

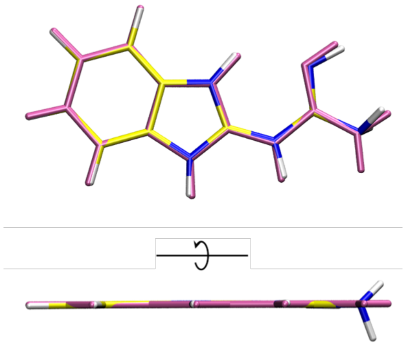

Figure S1. A molecule overlay of 2GBI (gbi2) compares the geometry-optimized structures from quantum mechanics (MP2/6-31G*) (colored in mauve) and the unmodified CGenFF force field (colored in blue and yellow). Poor assignment of initial atom types in the force field led to a pyramidal geometry of the -NH_2 group in the guanidine region.

The CHARMM force field parameters for gbi1 were developed using CGenFF.[1–4] The force field for gbi2 was generated from the CGenFF webserver, `https://cgenff.umaryland.edu/`, which performs atom typing and parameter/charge assignment by analogy. The resulting force field was modified with comparison to a reference structure from an *ab initio* QM MP2/6-31G*[5–9] geometry optimization. We compared the geometry-optimized structures from QM and the force field, then we conduct torsion scans around the dihedral angle labeled by the four highlighted atoms in Figure 1.

The original initial force field had a poor choice of assigned atom types for the nitrogen and hydrogen atoms of the terminal -NH_2 group in the guanidine region, leading to a minimized structure that is non-planar as shown in blue and yellow in Figure S1. The mauve structure shows the QM-optimized structure for reference. Changes were made to the CGenFF force field to redefine those two atom types going from a methylamine-like moiety to an imine-like moiety. We also lightly adjusted standard and dihedral angle parameters to improve the consistency of the MM geometry to the QM geometry.

Secondly, we computed energy profiles for torsion scans of the dihedral angle labeled in Figure 1. The QM result is plotted in red in Figure S2, and the MM result is shown as the blue markers. There is reasonable agreement between QM and the force field except for the regions around the minima (0°, 180°) and maxima (90°, 270°). The deviation at the maxima is expected to have negligible influence on our protein-bound simulations as the torsion scans are similar up to 8 kcal/mol, and the likelihood for conformational states to be occupied decreases dramatically at very high energy levels. Further investigation at the 180°

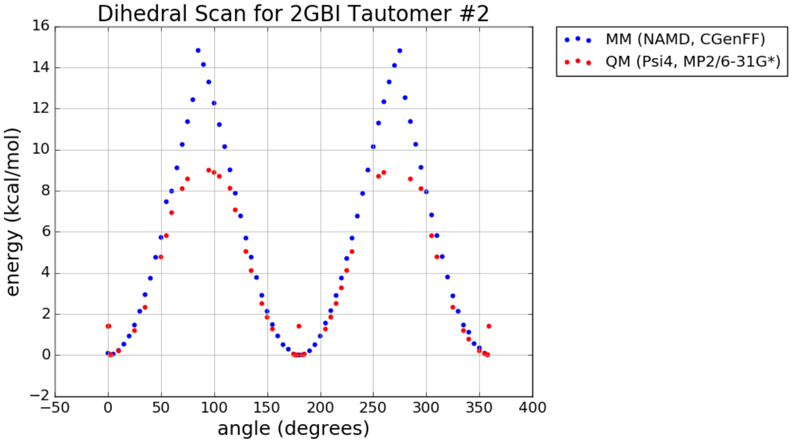

Figure S2. Overlay of dihedral scan energy profiles from the CGenFF force field (blue) and reference quantum mechanical data (red) for gbi2.

region (Figure S3) showed that the small plateau of QM energy (blue curve) reflects a steric interaction between two atoms of the guanidine region (red curve) which is not captured in classical MD simulations. For that reason, we did not attempt to further modify the dihedral parameters.

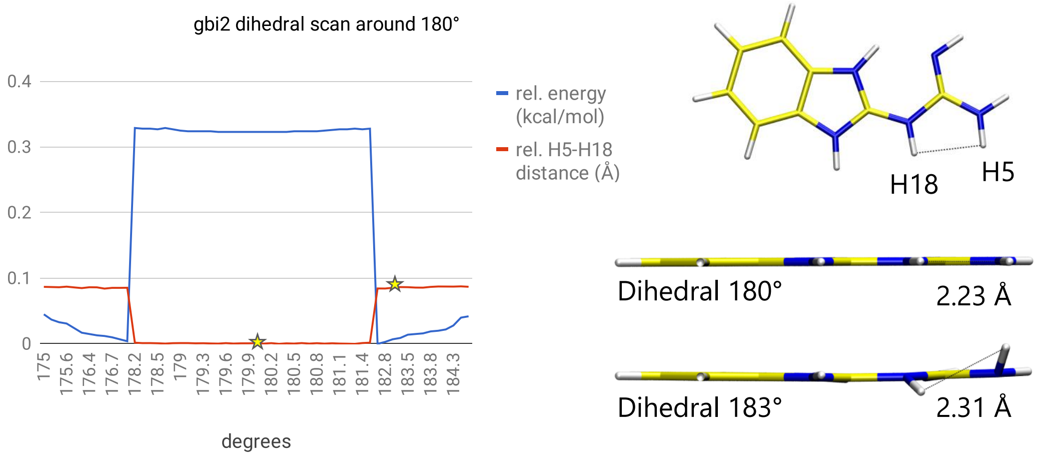

Figure S3. Focused dihedral scan around the 180° region of gbi2. We applied the QM method B3LYP-D3MBJ/def2-TZVP[10–14] for a more rigorous examination compared to the traditional MP2/6-31G* reference. The increase in relative dihedral energy is directly correlated to the H18-H5 distance, suggesting the influence of steric interactions.

Fixed dihedral angles in docking

The placement and orientation of 2GBI bound to Hv1 was explored using molecular docking with AutoDock Vina.[15] We impose planarity of 2GBI, rationalized by a planar optimized geometry and a higher energy barrier to rotate the dihedral angle (Figure 1) away from planar 180° (Figure S2 and Figure S4).

When we did not enforce a planar ligand during docking, this led to a variety of geometries for possible configurations of 2GBI in Hv1, including that shown in Figure S5. Docking tends to ignore internal strain energies in molecules so it is not necessarily unusual for it to generate highly strained candidate binding modes. Depending on the timescale for changes in binding mode, these can end up being retained throughout even lengthy MD simulations if the ligand is unable (often for steric reasons) to find something better.

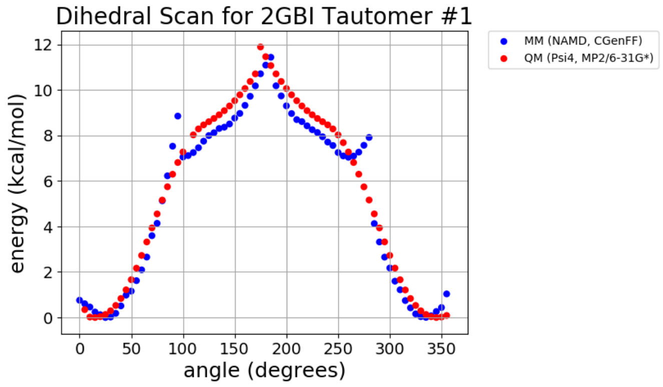

Figure S4. Overlay of dihedral scan energy profiles from the CGenFF force field (blue) and reference quantum mechanical data (red) for gbi1.

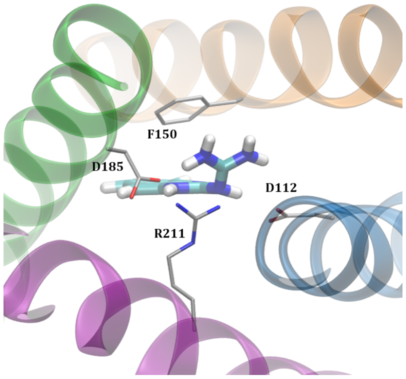

Figure S5. Illustrative example from docking and molecular dynamics for gbi1 bound to Hv1, viewing from extracellular end of channel. 2GBI is trapped in a strained configuration with its dihedral angle at 112° (cf. energy profile in Figure S4).

Pose selection and refinement with MD

A "reverse clustering" approach from fixed-planar docking results gave us distinct potential binding poses in the binding site. Each pose was assessed from putative protein-ligand interactions from Hong et al.[16] We also evaluated the fit of the ligand in the Hv1 binding region from MD simulations. Regarding protein-ligand interactions, we monitored the following distances: (1) between the center of the benzo moieties on 2GBI and F150, (2) between the central carbon atoms of the guanidine moieties on 2GBI and R211, and (3) between the closest imidazolic nitrogen atom on 2GBI with the closest oxygen atom on D112. The optimal pose showed relatively stable contact distances in each of these criteria as well as a stable protein backbone RMSD and stable ligand RMSD.

After docking, we assessed hydration of the ligand-bound protein pore in 20 ns of equilibrating MD simulations to verify that the pore was well-hydrated prior to free energy calculations (Figure S6). In Figure S7, we show our binding pose as in Figure 4 with additional protein residues labeled to compare to that of Chamberlin et al.[17]

dimension	lower bound	upper bound
x	A175, backbone N	A135, backbone C
y	L124, backbone O	E153, backbone O
z	H168, backbone CA	I127, backbone N

Table S1. Atom selections used to define coordinates of the bounding box to count pore waters in MD simulations following docking calculations.

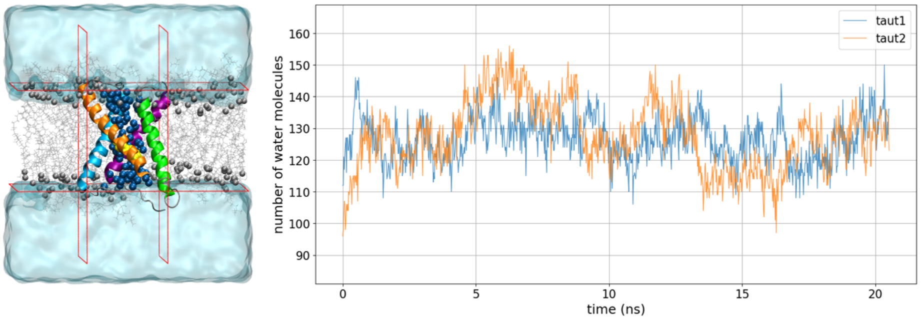

Figure S6. Count of water molecules within the pore of the 2GBI-bound Hv1 open state in equilibration simulations prior to free energy calculations. We counted waters within the rectangular prism illustrated on the left panel, where the box dimensions were based on the atom positions (following trajectory alignment on the Hv1 backbone) as listed in Table S1.

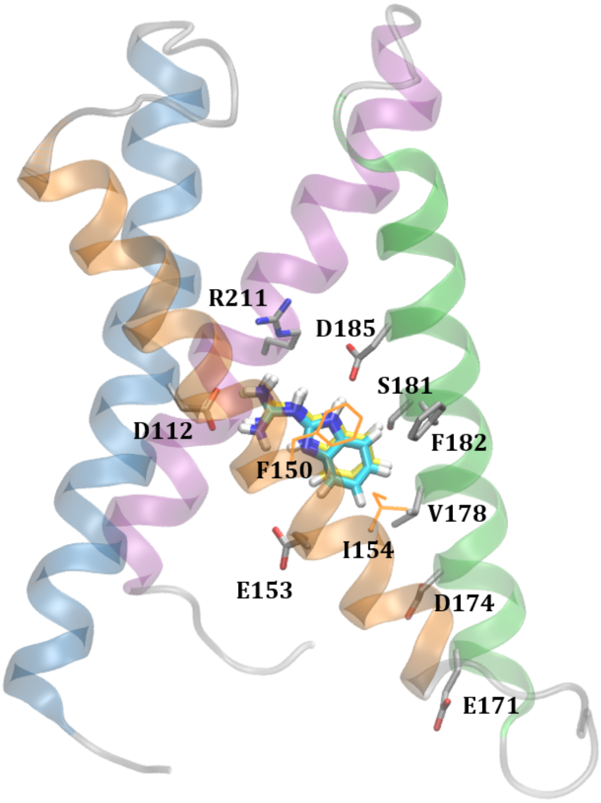

Figure S7. Our binding pose as in Figure 4 with additional protein residues labeled. The extracellular side of the channel is at the top of the figure. Select residues, namely E153, D174, and E171, comprise part of the binding pocket of 2GBI in *Ciona intestinalis* Hv1 as reported by Chamberlin et al. Interactions with these residues would lead to an intracellular-pointing ligand orientation which would preclude the protein-ligand interactions with S181 or R211 as proposed by Hong et al.

Analysis of protein-ligand interactions of bound ligand before and after D112E mutation

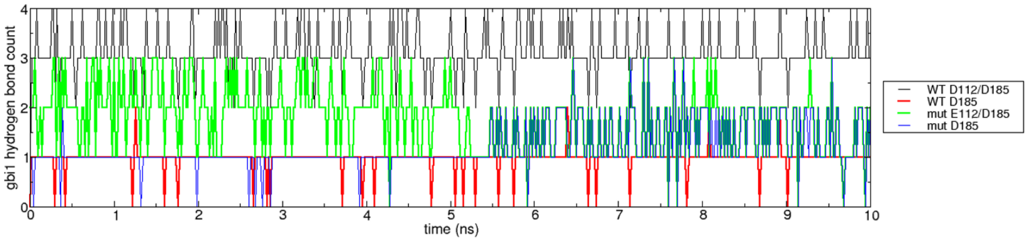

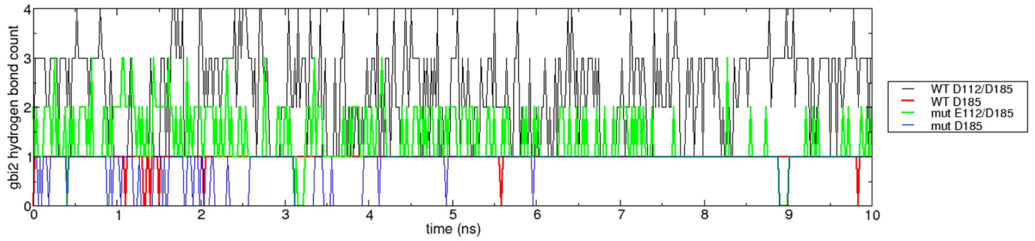

Figure S8. Hydrogen bond count between 2GBI and Hv1 residues 112 and 185 for 10 ns of simulation with WT Hv1 and with the D112E mutant of Hv1 for (**top**) gbi1 and (**bottom**) gbi2. The black and green lines represent the total hydrogen bond count between the ligand and D112/D185 before and after mutation, respectively. The red and blue lines represent the number of hydrogen bonds between 2GBI and only D185 before and after mutation, respectively. The hydrogen bond counts to both acidic residues are generally higher before D112E mutation (black vs. green) for both tautomers. The hydrogen bonds to D185 are slightly disrupted after mutation, especially for gbi2 (bottom; red vs. blue). There is an increase in hydrogen bonding interactions between gbi1 and D185 after around 5 ns as the ligand reorients as described in the main text (Figure 6).

Flat-bottom restraints for mutations involving Arg or Lys residues

The charge neutralization of flexible side chains during alchemical free energy calculation may lead to the loss of native electrostatic interactions with proximal side chains. This may result in artifactual configurations of the transforming side chain that adversely affects the calculated binding free energies (Figure 7). To address this issue, we used the `colvars` module of NAMD[18] to add flat-bottom restraints to arginine and lysine residues partaking in mutation. This imposes an energetic penalty when the variable (here, a distance) is below some threshold x_0 or above some threshold x_1. In between the thresholds, the potential is zero and no additional force is exerted. The distances defining the flat-bottom restraint parameters are shown in the following table:

mutation	atom of mutating side chain	atom of reference side chain	x_0: lower boundary (Å)	x_1: upper boundary (Å)
R208K	R208 CZ	D123 CG	3.0	8.5
R208K	R208 CZ	E119 CD	3.0	8.5
R208K	R208 CZ	E192 CD	3.0	8.5
R208K	K208 NZ	D123 CG	3.0	8.5
R208K	K208 NZ	E119 CD	3.0	8.5
R208K	K208 NZ	E192 CD	3.0	8.5
R211S	R211 CZ	D112 CG	3.5	7.0
R211S	R211 CZ	D185 CG	3.5	7.0
R211S	R211 CZ	F150 CG	6.0	10.5

Table S2. Atom labels and distance parameters used in flat-bottom restraints imposed in mutating Arg or Lys residues. Shaded rows represent restraints that were applied to the holo protein only.

The distance boundaries were chosen to be restrictive enough to prevent excessive side chain mobility, but also flexible enough

to allow conformational sampling.

A correction must be applied to the computed free energies to unbias the effects of the restraints. The thermodynamic cycle for correcting the relative binding free energies is depicted in Figure S9.

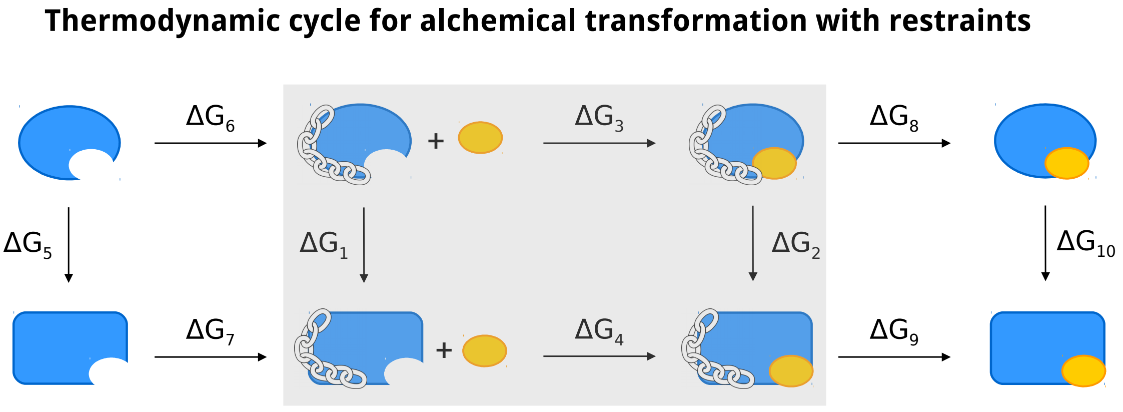

$$
\begin{aligned}
\Delta\Delta G_{expt} &= \Delta G_{10} - \Delta G_5 \\
\Delta G_6 + \Delta G_3 + \Delta G_8 + \Delta G_{10} &= \Delta G_5 + \Delta G_7 + \Delta G_4 + \Delta G_9 \\
\Delta G_{10} - \Delta G_5 &= \Delta G_7 + \Delta G_4 + \Delta G_9 - (\Delta G_6 + \Delta G_3 + \Delta G_8) \\
&= (\Delta G_7 - \Delta G_6) + (\Delta G_4 - \Delta G_3) + (\Delta G_9 - \Delta G_8) \\
&= (\Delta G_7 - \Delta G_6) + \underbrace{(\Delta G_2 - \Delta G_1)}_{\text{from FEP}} + (\Delta G_9 - \Delta G_8)
\end{aligned}
$$

Figure S9. Thermodynamic cycle for computing relative binding free energies in an alchemical transformation, modified to account for the addition of side chain restraints. The small orange oval represents the ligand, the large blue oval represents the wild type protein, and the blue rectangle represents the mutated protein. The shaded region represents the standard thermodynamic cycle, such as that shown in Figure 2, with the incorporation of flat-bottom restraints on the mutating side chain. The processes represented by each component are as follows: ΔG_1, alchemical free energy of mutation in apo protein (restrained); ΔG_2, alchemical free energy of mutation in holo protein (restrained); ΔG_3, artificial binding free energy of wild type protein (restrained); ΔG_4, artificial binding free energy of mutant protein (restrained); ΔG_5, alchemical free energy of mutation in apo protein (unrestrained); ΔG_6, free energy to add restraints to apo wild type protein; ΔG_7, free energy to add restraints to apo mutant protein; ΔG_8, free energy to remove restraints from holo wild type protein; ΔG_9, free energy to remove restraints from holo mutant protein; ΔG_{10}, alchemical free energy of mutation in holo protein (unrestrained). The effects of the side chain restraints can be removed from the computed relative binding free energy ($\Delta G_2 - \Delta G_1$) by the four additional terms of ΔG_7, $-\Delta G_6$, ΔG_9, and $-\Delta G_8$.

The free energy computations require eight additional simulations to estimate ΔG_7, $-\Delta G_6$, ΔG_9, and $-\Delta G_8$. Each of these terms is determined by calculating the distances between each restrained pair of side chains (the forward work), calculating the distances between each non-restrained pair of side chains (the reverse work), computing the associated energies from the flat-bottom potential, subsampling based on statistical inefficiency, then calculating the free energy difference from the BAR estimator.[19,20] To calculate the free energy to remove the restraints for correcting the relative binding free energies, the forward work represents the energy to remove the restraints from a restrained simulation, and the reverse work represents the energy to introduce restraints into a non-restrained simulation. For well-chosen restraint distances such that the side chain distances fall within the limits of the restraints even when no restraints are applied, the ΔG correction term goes to zero. As such, we defined our restraint distances so that the additional ΔG terms of the correction were zero.

Potential of mean force calculation for F182 rotation after V178A mutation

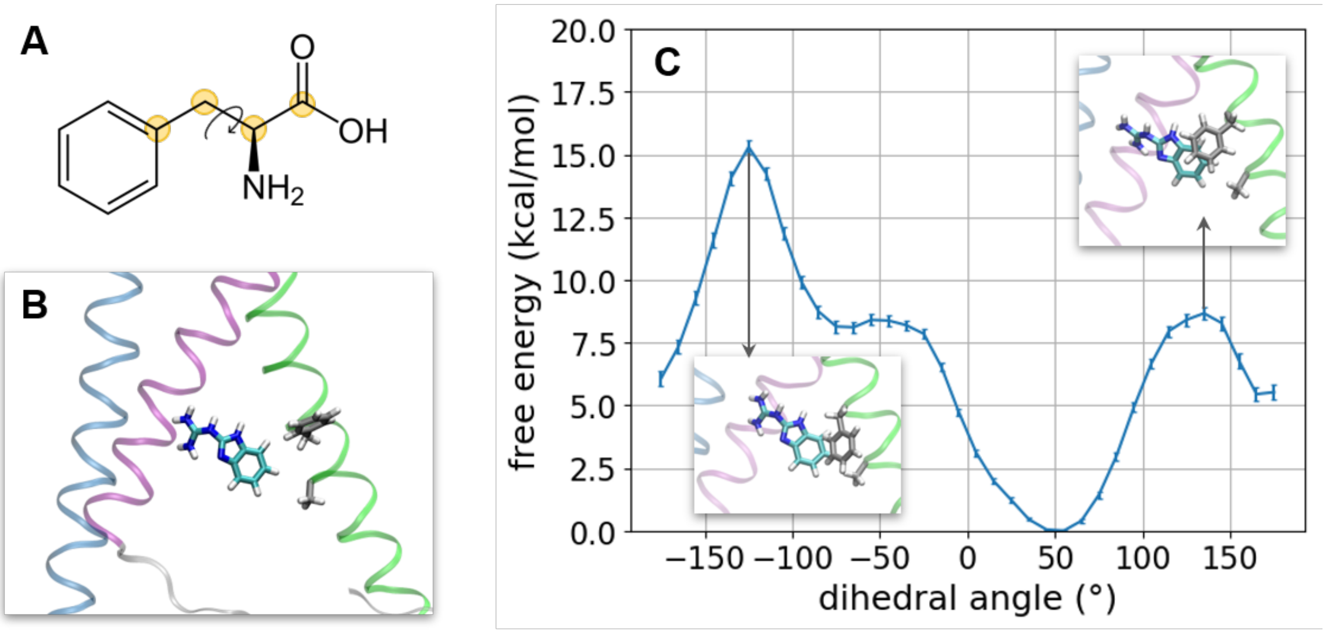

Figure S10. Consideration of potential ligand stabilization by reorientation of F182 after V178A mutation. (**A**) The highlighted four atoms define the dihedral angle of interest around which we consider rotation of the F182 side chain. (**B**) Representative snapshot from equilibrium MD simulations with F182 dihedral angle at 46°. S2 is not shown for clarity, and colors are consistent with those in Figure 3. This shows approximately the most energetically favorable configuration of this phenylalanine residue. (**C**) Potential of mean force for rotation of the F182 dihedral angle. Two insets show the position of F182 at -130° and 140°.

The V178A mutation is expected to contribute to a slightly more favorable 2GBI binding free energy relative to wild type Hv1 according to mutagenesis experiments. Our computed values suggested a slightly more unfavorable binding free energy. We examined the effect of secondary conformational changes in the protein that may result after the V178A mutation. Specifically, we focused on F182, which is in proximity to both V178 and 2GBI (we consider gbi1 only). We used umbrella sampling[21] to compute the potential of mean force (PMF) for rotation of the side chain, which could lead to better interactions with the bound ligand.

The dihedral angle, defined by the four atoms shown in Figure S10A, was restrained in increments of 10° from -180° to 180° using a harmonic potential with a force constant of 0.1 kcal/mol/deg^2. Each configuration was minimized for 5000 steps with the conjugate gradient algorithm, then run for 5 ns with the MD simulation settings as described in the main text. We used the MBAR algorithm[20] to generate the PMF and associated error bars. It does not appear that reorientation of F182 as considered here contributes to the favorable relative binding free energy of V178A.

References

1. Geragotelis, A. D. *et al.* Voltage-dependent structural models of the human Hv1 proton channel from long-timescale molecular dynamics simulations. *Under review* (2019).
2. Wood, M. L. *Investigating the Structure and Function of Ion Conducting Voltage Sensing Domains*. Ph.D. thesis, University of California, Irvine (2016). ProQuest ID: Wood_uci_0030D_13869. Merritt ID: ark:/13030/m5324hpp. Retrieved from https://escholarship.org/uc/item/9j86t5s7.
3. Vanommeslaeghe, K. & MacKerell, A. D. Automation of the CHARMM General Force Field (CGenFF) I: Bond Perception and Atom Typing. *J. Chem. Inf. Model.* **52**, 3144–3154, DOI: 10.1021/ci300363c (2012).
4. Vanommeslaeghe, K., Raman, E. P. & MacKerell, A. D. Automation of the CHARMM General Force Field (CGenFF) II: Assignment of Bonded Parameters and Partial Atomic Charges. *J. Chem. Inf. Model.* **52**, 3155–3168, DOI: 10.1021/ci3003649 (2012).
5. Head-Gordon, M., Pople, J. A. & Frisch, M. J. MP2 energy evaluation by direct methods. *Chem. Phys. Lett.* **153**, 503–506, DOI: 10.1016/0009-2614(88)85250-3 (1988).
6. Weigend, F., Häser, M., Patzelt, H. & Ahlrichs, R. RI-MP2: Optimized auxiliary basis sets and demonstration of efficiency. *Chem. Phys. Lett.* **294**, 143–152, DOI: 10.1016/S0009-2614(98)00862-8 (1998).
7. Frisch, M. J., Head-Gordon, M. & Pople, J. A. A direct MP2 gradient method. *Chem. Phys. Lett.* **166**, 275–280, DOI: 10.1016/0009-2614(90)80029-D (1990).
8. Frisch, M. J., Head-Gordon, M. & Pople, J. A. Semi-direct algorithms for the MP2 energy and gradient. *Chem. Phys. Lett.* **166**, 281–289, DOI: 10.1016/0009-2614(90)80030-H (1990).
9. Rassolov, V. A., Pople, J. A., Ratner, M. A. & Windus, T. L. 6-31G* basis set for atoms K through Zn. *J. Chem. Phys.* **109**, 1223–1229, DOI: 10.1063/1.476673 (1998).
10. Becke, A. D. Density-functional thermochemistry. III. The role of exact exchange. *J. Chem. Phys.* **98**, 5648–5652, DOI: 10.1063/1.464913 (1993).
11. Lee, C., Yang, W. & Parr, R. G. Development of the Colle-Salvetti correlation-energy formula into a functional of the electron density. *Phys. Rev. B* **37**, 785–789, DOI: 10.1103/PhysRevB.37.785 (1988).
12. Vosko, S. H., Wilk, L. & Nusair, M. Accurate spin-dependent electron liquid correlation energies for local spin density calculations: A critical analysis. *Can. J. Phys.* **58**, 1200–1211, DOI: 10.1139/p80-159 (1980).
13. Stephens, P. J., Devlin, F. J., Chabalowski, C. F. & Frisch, M. J. Ab Initio Calculation of Vibrational Absorption and Circular Dichroism Spectra Using Density Functional Force Fields. *J. Phys. Chem.* **98**, 11623–11627, DOI: 10.1021/j100096a001 (1994).
14. Weigend, F. & Ahlrichs, R. Balanced basis sets of split valence, triple zeta valence and quadruple zeta valence quality for H to Rn: Design and assessment of accuracy. *Phys. Chem. Chem. Phys.* **7**, 3297–3305, DOI: 10.1039/B508541A (2005).
15. Trott, O. & Olson, A. J. AutoDock Vina: Improving the speed and accuracy of docking with a new scoring function, efficient optimization, and multithreading. *J. Comput. Chem.* **31**, 455–461, DOI: 10.1002/jcc.21334 (2010).
16. Hong, L., Kim, I. H. & Tombola, F. Molecular determinants of Hv1 proton channel inhibition by guanidine derivatives. *Proc Natl Acad Sci USA* **111**, 9971, DOI: 10.1073/pnas.1324012111 (2014).
17. Chamberlin, A. *et al.* Hydrophobic plug functions as a gate in voltage-gated proton channels. *Proc Natl Acad Sci USA* **111**, E273, DOI: 10.1073/pnas.1318018111 (2014).
18. Fiorin, G., Klein, M. L. & Hénin, J. Using collective variables to drive molecular dynamics simulations. *Mol. Phys.* **111**, 3345–3362, DOI: 10.1080/00268976.2013.813594 (2013).
19. Bennett, C. H. Efficient estimation of free energy differences from Monte Carlo data. *J. Comput. Phys.* **22**, 245–268, DOI: 10.1016/0021-9991(76)90078-4 (1976).
20. Shirts, M. R. & Chodera, J. D. Statistically optimal analysis of samples from multiple equilibrium states. *J. Chem. Phys.* **129**, 124105, DOI: 10.1063/1.2978177 (2008).
21. Torrie, G. M. & Valleau, J. P. Nonphysical sampling distributions in Monte Carlo free-energy estimation: Umbrella sampling. *J. Comput. Phys.* **23**, 187–199, DOI: 10.1016/0021-9991(77)90121-8 (1977).
